# Neuromuscular blocking agents in patients with acute respiratory distress syndrome: a summary of the current evidence from three randomized controlled trials

**DOI:** 10.1186/cc12657

**Published:** 2013-06-19

**Authors:** VGM Pereira

**Affiliations:** 1 ABC Medical School (FMABC), Príncipe De Gales, Santo André, SP, Brazil

## Introduction

Acute respiratory distress syndrome (ARDS) is a potentially fatal disease with high mortality. Our aim was to summarize the current evidence for use of neuromuscular blocking agents (NMBA) in the early phase of ARDS.

## Methods

A systematic review and meta-analysis of publications between 1966 and 2012. The Medline and CENTRAL databases were searched for studies on NMBA in patients with ARDS. The meta-analysis was limited to: randomized controlled trials; adult human patients with ARDS or acute lung injury; and use of any NMBA in one arm of the study compared with another arm without NMBA. The outcomes assessed were: overall mortality, ventilator-free days, time of mechanical ventilation, adverse events, and changes in gas exchange, in ventilator settings, and in respiratory mechanics.

## Results

Three randomized controlled trials covering 431 participants were included. Patients treated with NMBA showed less mortality (risk ratio, 0.71 (95% CI, 0.55 to 0.90); number needed to treat, 1 to 7), more ventilator-free days at day 28 (*P *= 0.020), higher PaO_2 _to FiO_2 _ratios (*P *= 0.004), and less barotraumas (*P *= 0.030). The incidence of critical illness neuromyopathy was similar (*P *= 0.540).

## Conclusion

The use of NMBA in the early phase of ARDS improves outcome.

